# The Intracellular Distribution of the Small GTPase Rho5 and Its Dimeric Guanidine Nucleotide Exchange Factor Dck1/Lmo1 Determine Their Function in Oxidative Stress Response

**DOI:** 10.3390/ijms23147896

**Published:** 2022-07-18

**Authors:** Linnet Bischof, Franziska Schweitzer, Carolin C. Sterk, Jürgen J. Heinisch

**Affiliations:** Department of Genetics, Faculty of Biology/Chemistry, University of Osnabrueck, Barbarastrasse 11, D-49076 Osnabrueck, Germany; linnet.bischof@uni-osnabrueck.de (L.B.); fschweitzer@uni-osnabrueck.de (F.S.); csterk@uni-osnabrueck.de (C.C.S.)

**Keywords:** Rho-type GTPase, mitochondria, oxidative stress, membrane trapping

## Abstract

Rho5, the yeast homolog of human Rac1, is a small GTPase which regulates the cell response to nutrient and oxidative stress by inducing mitophagy and apoptosis. It is activated by a dimeric GEF composed of the subunits Dck1 and Lmo1. Upon stress, all three proteins rapidly translocate from the cell surface (Rho5) and a diffuse cytosolic distribution (Dck1 and Lmo1) to mitochondria, with translocation of the GTPase depending on both GEF subunits. We here show that the latter associate with mitochondria independent from each other and from Rho5. The trapping of Dck1-GFP or GFP-Lmo1 to the mitochondrial surface by a specific nanobody fused to the transmembrane domain (TMD) of Fis1 results in a loss of function, mimicking the phenotypes of the respective gene deletions, *dck1* or *lmo1*. Direct fusion of Rho5 to Fis1^TMD^, i.e., permanent attachment to the mitochondria, also mimics the phenotypes of an *rho5* deletion. Together, these data suggest that the GTPase needs to be activated at the plasma membrane prior to its translocation in order to fulfill its function in the oxidative stress response. This notion is substantiated by the observation that strains carrying fusions of Rho5 to the cell wall integrity sensor Mid2, confining the GTPase to the plasma membrane, retained their function. We propose a model in which Rho5 activated at the plasma membrane represses the oxidative stress response under standard growth conditions. This repression is relieved upon its GEF-mediated translocation to mitochondria, thus triggering mitophagy and apoptosis.

## 1. Introduction

Yeast cells have evolved to respond to drastic changes in their environment by appropriately changing their metabolism and/or gene expression. For this purpose, different signalling cascades have been installed which frequently involve the use of small GTPases as molecular switches. A subfamily of the latter is comprised by the Rho-type GTPases (for “Ras homology”), six of which have been described in *S. cerevisiae*, namely Rho1 to Rho5, in addition to Cdc42 [1,2]. They mediate diverse physiological adaptations, ranging from cell wall integrity (CWI) signalling (Rho1 [3]) to cell polarity establishment in budding and cell fusion during mating (Cdc42 [4]). The switch is turned on by binding of a GTP molecule, and off by its hydrolysis to GDP [5,6,7]. The interconversion between these states is aided by the action of guanine nucleotide exchange factors (GEFs), which facilitate the exit of GDP and its substitution by GTP due to the higher intracellular concentration of the latter, and GTPase activating proteins (GAPs), which stimulate the intrinsic hydrolytic activity, respectively [8,9]. After their lipid modification at a C-terminally conserved cysteine residue (CAAX-box), the GTPases associate with cellular membranes, where they exert their physiological functions [10]. Cytosolic trafficking in the inactive state can be aided by masking the lipid anchor through association with a GDP-dissociation inhibitor protein (GDI, Ref. [11]).

**Figure 1:**
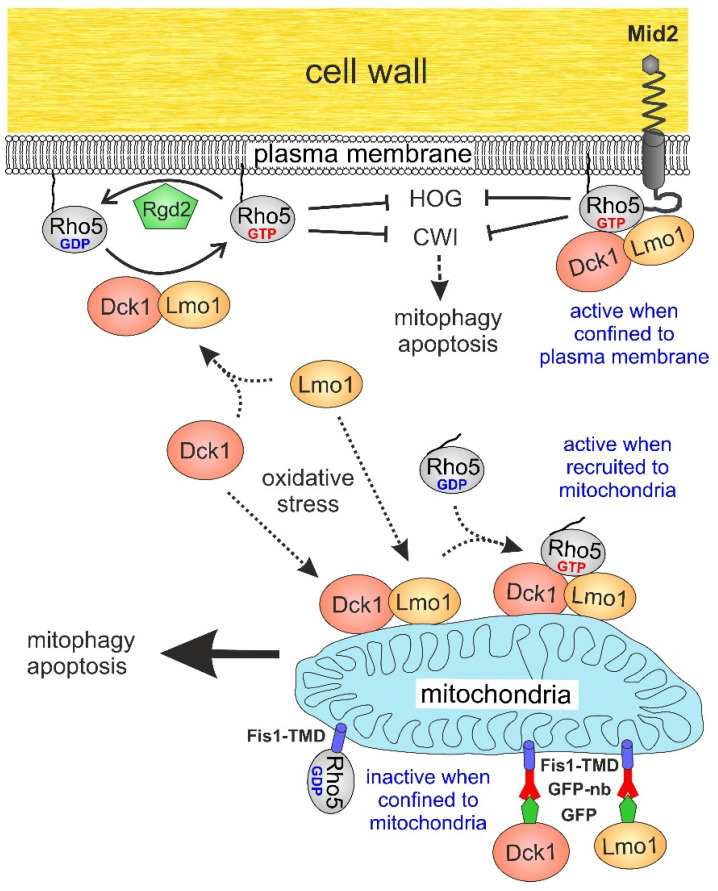
Proposed role of the intracellular distribution of Rho5 and its dimeric GEF Dck1/Lmo1 in the oxidative stress response. Rho5 is inactive in its GDP-bound and active in its GTP-bound state, which are interconverted by the help of a GTPase activating protein (GAP, Rgd2) and the dimeric GDP/GTP exchange factor (GEF, Dck1/Lmo1). Negative regulation (lines with bars) of the cell wall integrity pathway (CWI) and the high osmolarity glycerol pathway (HOG) is indicated for the active Rho5 associated with the plasma membrane by its lipid anchor (wavy line). A possible indirect effect of the CWI pathway on mitophagy and apoptosis (see discussion section) is symbolized by the dashed arrow. Dotted arrows show proposed routes of intracellular trafficking of the GTPase and its GEF subunits under different physiological conditions. Fusion constructs to confine Rho5 to either the plasma membrane through the CWI sensor Mid2, or to the mitochondrial outer membrane through the transmembrane domain (TMD) of Fis1, either directly or fused to a GFP nanobody (GFP-nb), constructed in this work are also indicated. Phenotypes regarding oxidative stress response are highlighted in blue print.

Rho5 was the last member of the subfamily described in *S. cerevisiae*, then as a negative regulator of CWI signalling [12]. Later studies implicated its function in a variety of other signalling processes, suggesting that it may be a central hub in their coordination (reviewed in [13]). Briefly, the synthetic lethality of a hyperactive *RHO5* allele with a *ste50* deletion provided evidence that the GTPase works as a negative regulator in the high osmolarity glycerol (HOG) pathway, which could be viewed as having an opposite function to CWI signalling, as it responds to high rather than low medium osmolarity [14]. Moreover, the observed synthetic lethalities of *rho5 ras2* and *rho5 sch9* deletions suggest a repressing function of the GTPase in nutrient starvation [15]. In addition to its proposed role as a repressor of these signalling pathways, Rho5 was also found to regulate the response to oxidative stress, as judged from the hyper-resistance of its deletion mutants to hydrogen peroxide [16]. In fact, the GTPase was found to rapidly translocate from the plasma membrane to mitochondria upon exposure of the cells to this agent, a process dependent on the presence of its dimeric GEF (guanine nucleotide exchange factor), which is composed of two subunits encoded by *DCK1* and *LMO1* (Figure 1; ref. [17]). 

In that work, a lack of either Rho5 or one of the GEF subunits was shown to drastically reduce mitophagy and apoptosis in *S. cerevisiae*, indicating that active Rho5 triggers these processes upon oxidative stress [17]. As the downstream MAP kinases in both the CWI and the HOG signalling pathway were shown to be required for mitophagy [18,19], it seems plausible that Rho5 at least partially acts by modulating their activity. A more direct role in mitophagy was also suggested from high-throughput screens, which identified Atg21 and Msp1, components of the mitophagy pathway and the outer mitochondrial membrane, respectively, as interaction partners of Rho5 [20].

Studies on the domain structure of Rho5 showed an unusual extension of 98 amino acids in the C-terminal half, which precedes the polybasic region (PBR) and the CAAX-box common in Rho-type GTPases [21]. All three regions were required for proper stress-induced translocation of yeast Rho5 [22]. Moreover, the trapping of GFP-tagged Rho5 to the mitochondrial surface in vivo by a specific nanobody rendered it non-functional with regard to oxidative stress, with phenotypes similar to the *rho5* deletion. It was thus proposed that the GTPase needs to be activated at the plasma membrane in order to fulfill its role in mitophagy and apoptosis [22].

Translocation of homologs of Rho5 and the two GEF subunits to mitochondria was also observed under oxidative and nutrient stress in the more respiratory yeast *K. lactis*, in which *Klrho5* deletions displayed pronounced morphological defects, in contrast to *S. cerevisiae* [23]. This is reminiscent of its human homolog Rac1, which, amongst a variety of cellular processes, regulates the dynamics of the actin cytoskeleton [24]. Like Rho5, Rac1 can be activated by the GEF DOCK180 in a complex with the ELMO protein, which are homologs of yeast Dck1 and Lmo1, respectively [25,26]. Malfunctions of Rac1 have been associated with serious diseases, including cancer and diabetes [27,28,29,30]. Consequently, yeast expression systems may provide excellent tools to study the molecular functions of the associated *RAC1* alleles [23].

How mitochondrial translocation of yeast Rho5 is achieved, and, more importantly, how the physiological functions of Rho5 are related to its subcellular distribution, remains to be elucidated. In this work, we investigated whether the GEF subunits depend on each other and on Rho5 to translocate under oxidative stress. To gain some insight into the in vivo importance of this trafficking, the three proteins were confined to different subcellular microdomains, i.e., the plasma membrane and the mitochondrial surface. Phenotypic analyses suggest that Rho5 exerts important functions in oxidative stress response when still associated with the plasma membrane, rather than exclusively after its translocation to mitochondria.

## 2. Results

### 2.1. The GEF Subunits Dck1 and Lmo1 Translocate to Mitochondria Independent from Each Other or Rho5

**Figure 2:**
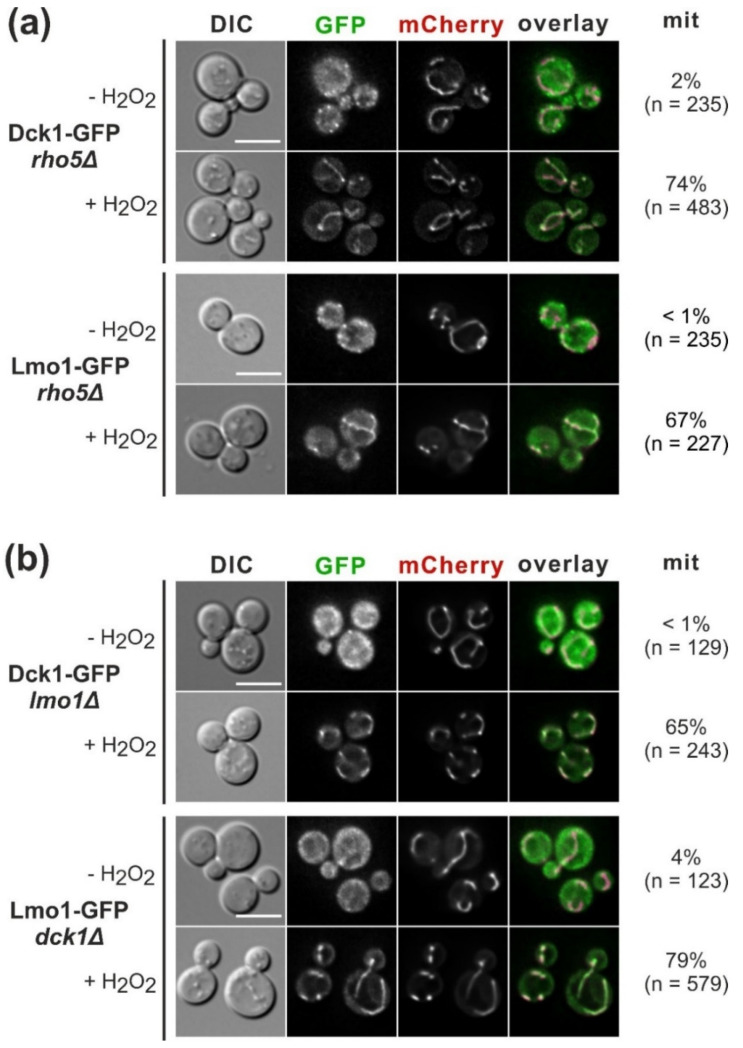
Requirements for translocation of Dck1 and Lmo1 to mitochondria under oxidative stress. (**a**) Dck1 and Lmo1 translocate independently from the presence of Rho5. (**b**) Dck1 and Lmo1 also translocate independent from each other. Strains expressing the GFP fusions of the indicated proteins from their native genomic loci were used to introduce a mitochondrial marker tagged with mCherry on a *CEN/ARS* plasmid (pJJH1408). Transformants were grown in selective minimal media. Representative images for each strain and condition are shown. When oxidative stress was applied by the addition of 4.4 mM hydrogen peroxide (+H_2_O_2_), fluorescence images were taken within less than 15 min with the indicated channels (GFP/mCherry), or using differential interference phase contrast (DIC). The percentage of cells displaying colocalization of the two fluorescence markers at mitochondria (mit) is given together with the number of total cells inspected (n). The size bars in the DIC images correspond to 5 µm, which is applicable to all images in the same panel. The strains employed were Dck1-GFP *rho5*Δ = HCSO20; Lmo1-GFP *rho5*Δ = HCSO25; Dck1-GFP *lmo1*Δ = HCSO26; and Lmo1-GFP *dck1*Δ = HCSO33. It should be noted that the C-terminal Lmo1-GFP fusion did not complement the phenotypic defects of a *lmo1* deletion, indicating that the tagged protein is not functional in vivo. However, a functional N-terminal GFP-Lmo1 fusion employed for the physiological studies in subsequent experiments was checked in the *dck1* and *rho5* deletion strains and confirmed the independent translocation.

While previous work revealed that oxidative stress-induced translocation of GFP-Rho5 to mitochondria depends on both subunits of the dimeric GEF Dck1/Lmo1 [17], whether the GEF subunits depend on the GTPase or on each other was not addressed. We thus checked the intracellular distribution of Dck1-GFP and Lmo1-GFP fusion proteins before and after the addition of hydrogen peroxide in different mutant backgrounds. Dck1- and Lmo1-GFP both displayed wild-type distributions when tested in a *rho5* deletion background under standard growth conditions and upon oxidative stress (Figure 2a). 

Interestingly, they were recruited to mitochondria independent from each other, as deletion of the gene encoding the other subunit (*LMO1* or *DCK1*, respectively) had no effect on stress-induced translocation (Figure 2b). Thus, stress signals presumably act on both Dck1 and Lmo1 to provoke their rapid translocation to mitochondria, for which neither the formation of the dimeric GEF, nor that of a trimeric complex with Rho5 is required.

### 2.2. Trapping of Dck1 or Lmo1 to Mitochondria Impedes Rho5 Function in the Oxidative Stress Response

**Figure 3:**
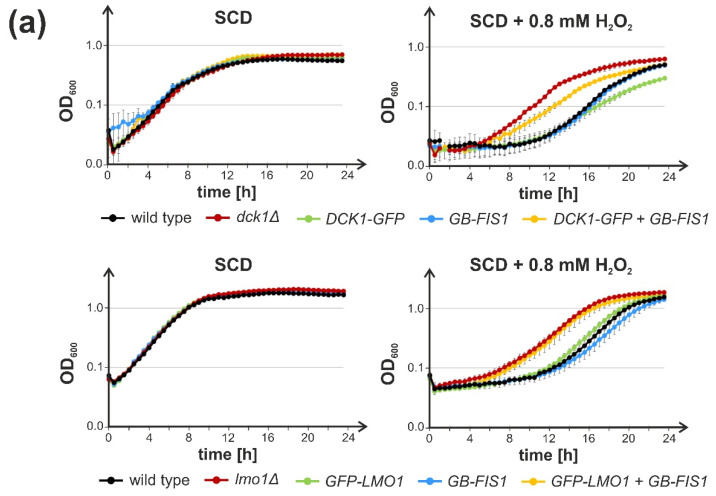

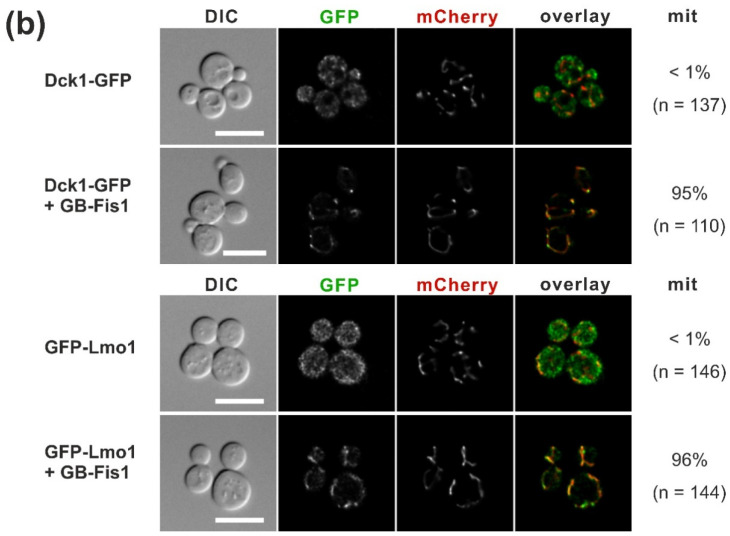
Trapping of GFP-tagged Dck1 and Lmo1 to mitochondria results in hyper-resistance towards oxidative stress. (**a**) Growth of strains producing Dck1-GFP (**upper** panel) or GFP-Lmo1 (**lower** panel) fusion proteins encoded at their native genomic loci in combination with a GFP binding nanobody attached to the mitochondrial surface (*GB-FIS1^TMD^*) in the absence (**left**) or presence (**right**) of hydrogen peroxide. Error bars give the standard deviations from growth recorded in duplicate for two independent strains, each, with the exception of the wild-type strain, for whom only one was measured in duplicate. Strains employed in the upper panel were: wild-type = HLBO20-4B; *dck1Δ* = HD56-5A/dck1ΔH1 and HD56-5A/dck1ΔKL6; *DCK1-GFP* = HLBO22-2A and HLBO22-4D; *GB-FIS1* = HLBO22-2D and HLBO22-9D; *DCK1-GFP + GB-FIS1* = HLBO22-3B and HLBO22-5B; and in the lower panel: wild-type = HLBO20-4B; *lmo1Δ* = LBO81 and HOD464-7B; *LMO1-GFP* = HCLO01-3B and HLBO19-3A; *GB-FIS1* = HCLO01-8B and HCLO01-27D; *LMO1-GFP + GB-FIS1* = HCLO01-15C and HCLO01-23D. (**b**) Dck1-GFP and GFP-Lmo1 are efficiently recruited to the mitochondrial surface by a nanobody fused to the transmembrane domain of Fis1. Fluorescence microscopy images were taken for cells grown in synthetic medium with 2% glucose as explained in the legend of Figure 2. An Idp1-mCherry fusion encoded at the native *IDP1* locus was used as a mitochondrial marker. Percentages of cells showing a colocalization of the tagged GEF subunits with mitochondria (mit) are given, calculated from the total number of cells inspected (n). Size bars correspond to 5 µm. The strains employed were *DCK1-GFP* = HLBO21-2A and HLBO21-10A; *DCK1-GFP + GB-FIS1 = *HLBO21-3D and HLBO21-6A*; GFP-LMO1 =* HLBO19-3B and HLBO19-5A*; GFP-LMO1 + GB-FIS1 =* HLBO19-1B and HLBO19-8C.

Given their independent translocation, we wondered whether the GEF subunits activate Rho5 at the mitochondrial surface to trigger the cell’s response to oxidative stress. To investigate this hypothesis, Dck1 and Lmo1 were trapped to the mitochondria, as previously exercised with GFP-Rho5, by using a “GFP binder” [22]. Therefore, the genes encoding the GEF subunits were tagged with GFP at their native loci, and combined by crossing and tetrad analyses with a strain carrying a specific GFP nanobody fused to the transmembrane domain of the mitochondrial outer membrane Fis1 protein. Since Lmo1-GFP constructs employed so far proved to be non-functional in subsequent physiological tests, i.e., they displayed a similar hyper-resistance towards hydrogen peroxide as the complete *lmo1* deletion (data not shown), the C-terminal tag was substituted by GFP attached to the N-terminal end. Neither Dck1-GFP nor GFP-Lmo1 alone or in combination with the GFP-binder affected growth of the respective strains under standard conditions (Figure 3a). 

Importantly, strains carrying only Dck1-GFP or GFP-Lmo1 were as sensitive to hydrogen peroxide as the wild-type, showing that both were fully functional. However, if efficiently trapped to the mitochondrial surface by the GFP-binder, as demonstrated by fluorescence microscopy (Figure 3b), a marked hyper-resistance of the respective strains was observed, mimicking the complete deletion in the case of GFP-Lmo1, and approaching the growth of the deletion strain for Dck1-GFP (Figure 3a). Consistent with the previous data on the trapping of the GFP-tagged GTPase itself, this suggested that in order to fulfill its physiological role in oxidative stress response, Rho5 has to be activated prior to reaching the mitochondria.

### 2.3. Attachment of Rho5 or its GEF Subunits to the Plasma Membrane Does Not Impair Its Repressor Function in Oxidative Stress Response

**Figure 4:**
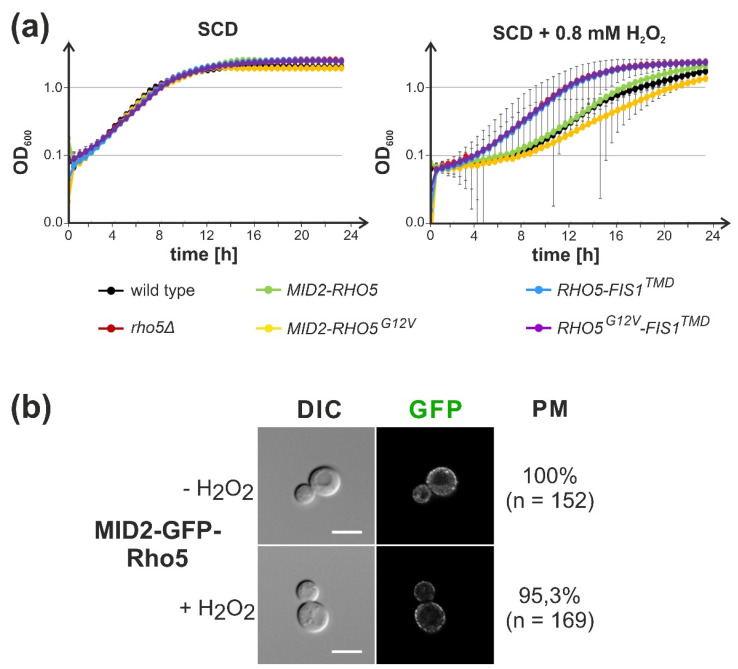
A fusion of Rho5 to the plasma membrane sensor Mid2 still functions in the oxidative stress response. (**a**) Growth of strains carrying the indicated fusions with *RHO5* either at the *MID2* locus (*MID2-RHO5, MID2-RHO5^G12V^*) or integrated with plasmid pJJH3024 at the *leu2-3,112* locus (*RHO5-FIS1^TMD^*) was followed under standard conditions (**left**) and under oxidative stress (**right**). Wild-type and *rho5* deletion strains were included as controls. Error bars give the standard deviations from growth recorded in duplicate for two independent strains, each, with the exception of *MID2-RHO5^G12V^*, for which only one strain was constructed. Strains employed were: wild type = FSO4-3A and FSO4-8A, rho5 = FSO4-3D and FSO4-8B, *MID2-RHO5* = HOD500-10A and HOD500-11B, *MID2-RHO5^G12V^ =* HFSO83, *RHO5-FIS1^TMD^* = LBO130 and LBO132, *RHO5^G12V^-FIS1^TMD^* = HOD529-2B and HOD529-2B. (**b**) Mid2-GFP-Rho5 fusions are confined to the plasma membrane with and without oxidative stress. Strain HOD512-3A was subjected to life cell fluorescence microscopy and representative bright-field (DIC, **left** panels) and fluorescence images (GFP channel; **right** panels) are shown. Percentages of cells with localization of the fusion protein at the plasma membrane (PM) were calculated from the total number of cells (n) observed.

In light of the results described above, we attempted to trap the GTPase in a similar approach by appropriate constructs with the GFP nanobody attached to different plasma membrane proteins. However, this did not yield satisfactory results, as fluorescence microscopy showed that a substantial amount of GFP-Rho5 still translocated to mitochondria upon application of oxidative stress (unpublished results from our laboratory). Therefore, the *RHO5* coding sequence was directly fused to the 3′end of the gene encoding the cell wall integrity sensor Mid2 at its native locus. The sensor was previously shown to reside in the plasma membrane in specific microdomains with a fairly uniform distribution, its C-terminus is exposed to the cytosol, and it is not subject to rapid endocytosis [31,32]. Strains carrying the *MID2-RHO5* fusion in conjunction with a *rho5* deletion grew like wild-type under normal conditions and in the presence of hydrogen peroxide (Figure 4). 

This suggested that Rho5 at the plasma membrane suffices to trigger the appropriate response to oxidative stress under these conditions, i.e., that translocation of Rho5 and its GEF to mitochondria, is not required. In contrast to previous findings on the pronounced the hyper-sensitivity of strains with an activated *RHO5^G12V^* allele towards oxidative stress [22], a pronounced difference as compared to the wild-type was found when the allele was fused with the *MID2* coding sequence. Vice versa, a direct fusion of Rho5 with the transmembrane domain of Fis1, which confines the GTPase to the outer mitochondrial membrane, rendered the strains as hyper-resistant towards hydrogen peroxide as a *rho5* deletion (Figure 4). This indicates a lack of GTPase function in the fusion protein, in accordance with previous data from trapping of Rho5 to mitochondria via a GFP nanobody [22].

## 3. Discussion

Rho5 in *S. cerevisiae* has been implicated, amongst other functions, in linking the oxidative stress response to mitochondrial turnover and apoptosis [16]. This notion is consistent with the rapid translocation of Rho5 and that of its dimeric GEF Dck1/Lmo1 to mitochondria upon exposure to hydrogen peroxide [17]. We here provided evidence that the translocation occurs for each individual GEF subunit, independent from each other and Rho5. Previous models proposed that the trimeric complex between the GTPase and its GEF could move as an entity from the plasma membrane to the mitochondrial surface [17,22], analogous to the complex suggested to be formed by the human homolog Rac1 with the DOCK180/ELMO [25]. The independent translocation of the GEF subunits observed here points to an alternative mechanism in which the GEF dimer could first be assembled at the mitochondrial surface and only then recruit Rho5. Such a sequence has been suggested for the Rab-GTPase Ypt7 involved in vesicle transport, which is recruited to and activated by its GEF associated with the target membrane [33]. In this context, human Rac1 also associates with various subcellular compartments, frequently mediated by its interaction with a plethora of specific GEFs (reviewed in [34]). It would therefore be interesting to determine the exact timing of the appearance of Dck1, Lmo1, and Rho5 at yeast mitochondria upon exposure to oxidative stress, or if they indeed travel together as a trimeric complex. Given that the translocation occurs within a matter of seconds [17], this may be challenging.

More importantly, we here addressed the physiological function of the intracellular distribution of the dimeric GEF by trapping either subunit to the mitochondrial surface through a GFP-tag and the corresponding nanobody. This rendered the strains hyper-resistant to oxidative stress, mimicking the phenotype of *dck1* and *lmo1* deletions. Similarly, confining Rho5 to the outer mitochondrial membrane by fusion with the transmembrane domain of Fis1 left the GTPase non-functional, showing the same hyper-resistance as a complete *rho5* deletion. These findings are consistent with previous data obtained from the nanobody-mediated trapping of GFP-Rho5 to mitochondria and support the notion that the GTPase needs to be activated at the plasma membrane prior to its translocation [22]. In contrast, the fusion of Rho5 to the plasma membrane sensor Mid2 did not affect the cell response to oxidative stress under the conditions tested herein. It should be noted that Mid2 is known to accumulate in membrane microdomains, which would cause an increased local concentration of the fused GTPase [31], in analogy to its human homolog, where local clustering of Rac1 was proposed as a mechanism of activation [35].

How is the localization and activity of Rho5 related to the oxidative stress response? The activated plasma membrane-bound Rho5 is believed to repress various signalling pathways, including CWI [12] and HOG [16]. In this context, upon exposure to hydrogen peroxide the CWI pathway was shown to transmit a signal generated by its sensors to the downstream MAP kinase Slt2 [36]. The kinase phosphorylates Cnc1, a cyclin regulating the cyclin-dependent kinase Cdk8, which triggers the nuclear exit of Cnc1. The latter apparently serves two functions: (i) it represses stress responsive genes in the nucleus in association with the mediator complex; and (ii) upon its expulsion into the cytosol, it associates with mitochondria and leads to their fission, mitophagy, and cell death [37]. However, it should be noted that the latter effect was observed under conditions of nitrogen starvation rather than with oxidative stress. Nevertheless, a lack of active Rho5 at the plasma membrane would be expected to increase CWI signalling, resulting in phosphorylation and nuclear exit of Cnc1, which could trigger mitophagy and apoptosis. How this could be related to a recent report on the involvement of the CWI sensor Mtl1 in the induction of autophagy and mitophagy upon glucose starvation during diauxic shift [38] also remains to be investigated.

In addition to these rather indirect actions of Rho5, we believe that its direct association with mitochondria also triggers oxidative stress-induced mitophagy and apoptosis. This requires activation of the GTPase prior to its translocation, as indicated by the null phenotypes of trapping either of the GEF subunits or Rho5 itself to the mitochondrial surface. On the other hand, a similar trapping of the activated GFP-Rho5^G12V^ variant restored sensitivity towards hydrogen peroxide to wild-type levels, whereas the untagged hyperactive GTPase was hyper-sensitive [22]. The fact that confining the hyper-active variant to the plasma membrane with the Mid2-Rho5^G12V^ fusion did not markedly increase sensitivity to oxidative stress as compared to the wild-type suggests that the intracellular trafficking of Rho5 also plays an important role.

## 4. Materials and Methods

### 4.1. Strains and Growth Conditions

Yeast strains employed and their genotypes are listed in Table 1. All strains derived from the HD56-5A and its isogenic diploid DHD5, which are closely related to the CEN.PK background [39,40]. 

The yeast cell culture and genetic techniques followed standard procedures [41]. Rich medium (YEPD) contained 1% yeast extract, 2% Bacto peptone (Difco Laboratories Inc., Detroit, MI, USA), and 2% glucose. Synthetic media were prepared as described in [41], with the omission of amino acids or bases as required for selection of plasmids or deletion markers and 2% glucose (SCD). For selection of the *kanMX* marker, 100 mg/L of G418 were added to the medium after sterilization. 

Growth curves were obtained in 100 µL cultures in 96 well plates, in SCD with or without hydrogen peroxide as indicated, and recorded in a Varioscan Lux plate reader (Thermo Scientific, Bremen, Germany) as described previously [22].

For tetrad analyses, diploid strains were grown to stationary phase in liquid YEPD, collected by centrifugation and dropped onto 1% potassium acetate plates for sporulation at 30 °C. After two to three days and microscopic inspection for ascus formation, a sample of each culture was resuspended in 100 µL of sterile water and 4 µL of Zymolyase 100T (10 mg/mL) was added, followed by 7–10 min incubation at room temperature. 15 µL of the suspension was streaked out onto a YEPD plate and spores were segregated using a Singer MSM400 micromanipulator (Singer Instruments, Somerset, UK). Plates were incubated for 4 days at 30 °C, and colony formation was documented by scanning. Scanned images were adjusted for brightness and contrast using Corel Photo Paint with the same settings for the entire plate prior to compilation of sections into the final figures.

For manipulations in *E. coli*, strain DH5α was employed with standard media as described previously [32].

### 4.2. Construction of Plasmids, Deletion Mutants and Gene Tagging

Wild-type genes from *S. cerevisiae* were obtained by PCR using appropriate oligonucleotides with restriction sites and genomic DNA of strain DHD5 or its derivatives as templates. Deletion strains and gene fusions with specific tags were obtained by one-step gene replacement techniques [42] by adding 45-50 bp of flanking sequences homologous to the target gene with appropriate oligonucleotides used for PCR amplification. For selection of in vivo recombinants, either the *kanMX* or the *SkHIS3* cassette from the Longtine collection [43], *SpHIS5* from pUG27, or *KlLEU2* from pUG73 were employed [44]. GFP tags were amplified from pJJH1619 (GFP-kanMX) or pJJH1620 (GFP-SkHIS3); mCherry tags from pJJH1524 (mCherry-SkHIS3), described in [23]. As a mitochondrial marker for fluorescence microscopy, either a genomic *IDP1-mCherry* fusion [22] was introduced by crossing with the appropriate strains and tetrad analysis, or plasmid pJJH1408 [17] was employed, which encodes a fusion of the Cox4-mitochondrial signal sequence with mCherry. 

For integration at the *leu2-3,112* locus, constructs were subcloned into the vector YIplac128 [45], and the resulting plasmids were linearized by digestion with BstEII prior to transformation and selection for leucine prototrophy. Proper integration was confirmed by PCR. Specifically, pLAO12 (*PFK2p-GB-FIS1^TMD^*) was used to integrate the GFP-nanobody construct, and pJJH3024 (*RHO5-FIS1^TMD^*) and pJJH3096 (*RHO5^G12V^-FIS1^TMD^*) for integration of the respective *RHO5* alleles fused to the mitochondrial transmembrane domain coding sequence.

All PCR-generated fragments were verified by Sanger sequencing (Seqlab, Göttingen, Germany). Maps and sequences of all plasmids and modifications of genomic loci are available upon request.

### 4.3. Fluorescence Microscopy

The setup used for the fluorescence microscopy consisted of a Zeiss Axioplan 2 (Carl Zeiss, Jena, Germany) equipped with a 100× alpha-Plan Fluor objective (NA 1.45) and differential-interference contrast. Sample handling and image processing have been described in detail in [17].

## 5. Conclusions

In conclusion, we assume that the GTPase has a dual role with regard to the oxidative stress response: while at the plasma membrane it represses the expression of stress-responsive nuclear genes through the CWI/Cnc1 relay under standard growth conditions. When stressed by hydrogen peroxide, Rho5 dissociates from the plasma membrane and thus relieves repression, priming the cells for mitophagy and apoptosis. The association of Rho5 with its GEF at the mitochondrial surface would then further promote the path to death. Further experiments to test the fusions obtained herein for their effect on mitophagy and apoptosis under strong oxidative stress are therefore required and currently in progress.

## Figures and Tables

**Table 1:** Strains constructed in this work.

Strain Name	Genotype ^1^
FSO4-3A	*MATalpha ura3-52 his3-11,15 leu2-3,112*
FSO4-3D	*MATalpha ura3-52 his3-11,15 leu2-3,112 rho5::kanMX*
FSO4-8A	*MATa ura3-52 his3-11,15 leu2-3,112*
FSO4-8B	*MATa ura3-52 his3-11,15 leu2-3,112 rho5::kanMX*
HCLO01-3B	*MATa ura3-52 his3-11,15 leu2-3,112 GFP-LMO1-KlLEU2*
HCLO01-8B	*MATalpha ura3-52 his3-11,15 leu2-3,112::pLA012 [GB-FIS1^TMD^]*
HCLO01-15C	*MATa ura3-52 his3-11,15 leu2-3,112::pLA012 [GB-FIS1^TMD^]* *GFP-LMO1-KlLEU2*
HCLO01-23D	*MATa ura3-52 his3-11,15 leu2-3,112::pLA012 [GB-FIS1^TMD^] GFP-LMO1-KlLEU2*
HCLO01-27D	*MATalpha ura3-52 his3-11,15 leu2-3,112::pLA012 [GB-FIS1^TMD^]*
HCSO20	*MATalpha ura3-52 his3-11,5 leu2-3,112 rho5::kanMX* *DCK1-3GFP-SkHIS3*
HCSO25	*MATa ura3-52 his3-11,5 leu2-3,112 rho5::kanMX LMO1-3GFP-SkHIS3*
HCSO26	*MATalpha ura3-52 his3-11,5 leu2-3,112 lmo1::kanMX* *DCK1-3GFP-SkHIS3*
HCSO33	*MATalpha ura3-52 his3-11,5 leu2-3,112 dck1::kanMX* *LMO1-3GFP-SkHIS3*
HD56-5A/ dck1ΔH1	*MATalpha ura3-52 his3-11,15 leu2-3,112 dck1::SpHIS5*
HD56-5A/ dck1ΔKL6	*MATalpha ura3-52 his3-11,15 leu2-3,112 dck1::KlLEU2*
HFSO83	*MATalpha ura3-52 his3-11,15 leu2-3,112 rho5:kanMX MID2-RHO5^G12V^::SkHIS3*
HLBO19-1B	*MATalpha ura3-52 his3-11,15 leu2-3,112::pLA012 [GB-FIS1^TMD^]* *GFP-LMO1-KlLEU2 IDP1-mCherry-kanMX*
HLBO19-3A	*MATalpha ura3-52 his3-11,5 leu2-3,112 GFP-LMO1-KlLEU2*
HLBO19-3B	*MATa ura3-52 his3-11,5 leu2-3,112 GFP-LMO1-KlLEU2* *IDP1-mCherry-kanMX*
HLBO19-5A	*MATalpha ura3-52 his3-11,5 leu2-3,112 GFP-LMO1-KlLEU2* *IDP1-mCherry-kanMX*
HLBO19-8C	*MATa ura3-52 his3-11,15 leu2-3,112::pLA012 [GB-FIS1^TMD^]* *GFP-LMO1-KlLEU2 IDP1-mCherry-kanMX*
HLBO20-4B	*MATa ura3-52 his3-11,15 leu2-3,112*
HLBO21-2A	*MATa ura3-52 his3-11,15 leu2-3,112 DCK1-3GFP-SpHIS5* *IDP1-mCherry-kanMX*
HLBO21-3D	*MATa ura3-52 his3-11,15 leu2-3,112::pLA012 [GB-FIS1^TMD^]* *DCK1-3GFP-SpHIS5* *IDP1-mCherry-kanMX*
HLBO21-6A	*MATalpha ura3-52 his3-11,15 leu2-3,112::pLA012 [GB-FIS1^TMD^]* *DCK1-3GFP-SpHIS5* *IDP1-mCherry-kanMX*
HLBO21-10A	*MATalpha ura3-52 his3-11,15 leu2-3,112 DCK1-3GFP-SpHIS5* *IDP1-mCherry-kanMX*
HLBO22-2A	*MATa ura3-52 his3-11,15 leu2-3,112 DCK1-3GFP-SpHIS5*
HLBO22-2D	*MATalpha ura3-52 his3-11,15 leu2-3,112::pLA012 [GB-FIS1^TMD^]*
HLBO22-3B	*MATalpha ura3-52 his3-11,15 leu2-3,112::pLA012 [GB-FIS1^TMD^]* *DCK1-3GFP-SpHIS5*
HLBO22-4D	*MATalpha ura3-52 his3-11,15 leu2-3,112 DCK1-3GFP-SpHIS5*
HLBO22-5B	*MATa ura3-52 his3-11,15 leu2-3,112::pLA012 [GB-FIS1^TMD^]* *DCK1-3GFP-SpHIS5*
HLBO22-9D	*MATa ura3-52 his3-11,15 leu2-3,112::pLA012 [GB-FIS1^TMD^]*
HOD464-7B	*MATa ura3-52 his3-11,15 leu2-3,112 lmo1::kanMX*
HOD500-10A	*MATa ura3-52 leu2-3,113 his3-11,15 rho5::kanMX MID2-RHO5-SkHIS3*
HOD500-11B	*MATalpha ura3-52 leu2-3,113 his3-11,15 rho5::kanMX* *MID2-RHO5-SkHIS3*
HOD512-3A	*MATa ura3-52 his3-11,15 leu2-3,112 MID2-GFP-RHO5-SkHIS3*
HOD529-2B	*MATa ura3-52 his3-11,15 leu2-3,112::pJJH3096 [LEU2-RHO5^G12V^-FIS1^TMD^] rho5::kanMX*
HOD529-5C	*MATalpha ura3-52 his3-11,15 leu2-3,112::pJJH3096 [LEU2-RHO5^G12V^-FIS1^TMD^] rho5::kanMX*
LBO81	*MATa ura3-52 his3-11,15 leu2-3,112 lmo1::KlURA3*
LBO130	*MATalpha ura3-52 his3-11,15 leu2-3,112::pJJH3024 [RHO5-FIS1^TMD^] rho5::kanMX*
LBO132	*MATa ura3-52 his3-11,15 leu2-3,112::pJJH3024 [RHO5-FIS1^TMD^] rho5::kanMX*

^1^ All strains employed are segregants from isogenic crosses derived from DHD5 [39].

## Data Availability

Not applicable.

## Abbreviations

*CEN/ARS*, centromere and autonomously replicating sequence of yeast plasmids with low copy numbers; CWI, Cell Wall Integrity pathway; DIC, Differential Interference Phase Contrast; GAP, GTPase Activating Protein; GDI, GDP Dissociation Inhibitor; GEF, Guanine nucleotide Exchange Factor; GFP-nb, GFP nanobody (also GB for GFP binder); HOG, High Osmolarity Glycerol pathway; PBR, Poly-Basic Region; *RHO5^G12V^*, allele of the yeast *RHO5* gene encoding an activated variant of the GTPase; SCD, synthetic complete medium with glucose; YEPD, rich medium with glucose; TMD, Trans-Membrane Domain.
